# Supplementary material for: Effect of Maternal Docosahexaenoic Acid (DHA) Supplementation on Offspring Neurodevelopment at 12 Months in India: A Randomized Controlled Trial
Source: Nutrients. 2020 Oct 3;12(10):3041. doi: 10.3390/nu12103041 (PMC7600740; doi:10.3390/nu12103041)
Supplement: Supplementary file 1 [file nutrients-12-03041-s001.pdf]

Supplementary tables:

eTable 1: Comparison of baseline characteristics those who completed the study and those who did not.

	Completed the study	Randomized but did not complete the study	p value
	N = 863	N = 94	
Maternal age (years), mean ± SD	23·5 ± 3·6	24·0 ± 3·7	0·22
Gestational age (weeks), median (p25, p75)	15·0 (12·0, 18·0)	14·5 (11·0, 18·0)	0·61
Primigravida, n (%)	346 (40·1%)	40 (42·6%)	0·64
Education, n (%)			
College graduated and above	156 (18·1%)	14 (14·9%)	0·62
High school/Secondary	681 (78·9%)	76 (80·9%)	
Employed, n (%)	209 (24·2%)	14 (14·9%)	0·04
Household income (>Rs 20,000), n (%)	101 (11·7%)	11 (11·7%)	0·50
Dietary Habits-Vegetarian, n (%)	73 (15·3%)	87 (18·2%)	0·71
Anthropometric Measurements (Mother)			
Height (cm), mean ± SD	153·9 ± 5·6	155·0 ± 5·5	0·08
Weight (Kg), mean ± SD	48·8 ± 8·7	49·4 ± 8·8	0·55
BMI (Kg/m2), mean ± SD	20·6 ± 3·5	20·6 ± 3·8	0·88
MUAC (cm), mean ± SD	24·3 ± 3·0	24·8 ± 3·3	0·14
Maternal Hb (gm%), mean ± SD	11·1 ± 1·3	11·1 ± 1·4	0·86

Data is presented as mean ± standard deviation or median (interquartile interval) or number (%)
BMI: Body mass index; MUAC: Mid upper arm circumference; Hb: Haemoglobin

eTable 2: Listing of Serious adverse events by group

SAE	DHA	Placebo	Total
Abortions	1	0	1
Abruptio Placenta	1	0	1
Congenital Anomalies	2	2	4
Fresh Still Birth (FSB)	4	4	8

Infant death	2	0	2
Macerated Still Birth (MSB)	3	2	5
Maternal Death	1	0	1
Medical Termination of Pregnancy (MTP)	0	1	1
Neonatal Death and Early Neonatal Death	2	0	2
Total	16	9	25

Congenital Anomalies: Supracardiac Total Anomalous Venous Connection-TAPAC; Ventricular Septal Defect; Congenital Talipes Equinovarus**;** Left sided Syndactyly of fingers and toes
Note: *3 SAE cases completed the study*

eTable 3: Primary Outcome (Per protocol analysis)

At 12th Month DQ score	DHA Mean (SD)	Placebo Mean (SD)	Difference* (95% CI)	p value
ITT	96·6 (12·1)	97·1 (13·0)	0·46 (-1·23, 2·14)	0·60
Per protocol	97·1 (11·7)	98·4 (12·3)	1·23 (-0·58, 3·05)	0·18

ITT principle [Group A n=433, Group B: n=430]; Per protocol definition-All infants who had a valid DQ score (±4 weeks) & Mother's compliance rate more than 80% & 48 protocol deviation cases [Total n=679, Group A n=345, Group B: n=334]

**Placebo minus DHA*

eTable 4: CONSORT 2010 checklist of information to include when reporting a randomised trial

**Section/ Topic**	**Item No**	**Checklist item**	**Reported on page No**
**Title and abstract**			
	1a	Identification as a randomized trial in the title	p.1
	1b	Structured summary of trial design, methods, results, and conclusions (for specific guidance see CONSORT for abstracts)	p. 3
**Introduction**			

Background and objectives	2a	Scientific background and explanation of rationale	p. 4-5
	2b	Specific objectives or hypotheses	p. 2, 5
**Materials and Methods**			
Trial design	3a	Description of trial design (such as parallel, factorial) including allocation ratio	p. 5
	3b	Important changes to methods after trial commencement (such as eligibility criteria), with reasons	No changes made
Participants	4a	Eligibility criteria for participants	p. 6
	4b	Settings and locations where the data were collected	p. 6-7
Interventions	5	The interventions for each group with sufficient details to allow replication, including how and when they were actually administered	p. 7-8
Outcomes	6a	Completely defined pre-specified primary and secondary outcome measures, including how and when they were assessed	p.8-9
	6b	Any changes to trial outcomes after the trial commenced, with reasons	NA
Sample size	7a	How sample size was determined	p. 10
	7b	When applicable, explanation of any interim analyses and stopping guidelines	NA
Randomisation:			
Sequence generation	8a	Method used to generate the random allocation sequence	p. 7
	8b	Type of randomisation; details of any restriction (such as blocking and block size)	p. 7

Allocation Concealment mechanism	9	Mechanism used to implement the random allocation sequence (such as sequentially numbered containers), describing any steps taken to conceal the sequence until interventions were assigned	p. 7
Implementation	10	Who generated the random allocation sequence, who enrolled participants, and who assigned participants to interventions	p. 7 -8
Blinding	11a	If done, who was blinded after assignment to interventions (for example, participants, care providers, those assessing outcomes) and how	p. 7
	11b	If relevant, description of the similarity of interventions	NA
Statistical methods	12a	Statistical methods used to compare groups for primary and secondary outcomes	p. 10-11
	12b	Methods for additional analyses, such as subgroup analyses and adjusted analyses	p. 11
**Results**			
Participant flow (a diagram is strongly recommended)	13a	For each group, the numbers of participants who were randomly assigned, received intended treatment, and were analysed for the primary outcome	p. 13 (Consort)
	13b	For each group, losses and exclusions after randomisation, together with reasons	p. 13 (Consort)
Recruitment	14a	Dates defining the periods of recruitment and follow-up	p. 11 and 16
	14b	Why the trial ended or was stopped	NA
Baseline data	15	A table showing baseline demographic and clinical characteristics for each group	(Table 1)

Numbers analysed	16	For each group, number of participants (denominator) included in each analysis and whether the analysis was by original assigned groups	Tables 1-4
Outcomes and estimation	17a	For each primary and secondary outcome, results for each group, and the estimated effect size and its precision (such as 95% confidence interval)	Table 4
	17b	For binary outcomes, presentation of both absolute and relative effect sizes is recommended	NA
Ancillary analyses	18	Results of any other analyses performed, including subgroup analyses and adjusted analyses, distinguishing pre-specified from exploratory	Figure 2
Harms	19	All-important harms or unintended effects in each group (for specific guidance see CONSORT for harms)	Supplementary eTable 3
**Discussion**			
Limitations	20	Trial limitations, addressing sources of potential bias, imprecision, and, if relevant, multiplicity of analyses	p. 17-19
Generalisability	21	Generalisability (external validity, applicability) of the trial findings	p. 17-19
Interpretation	22	Interpretation consistent with results, balancing benefits and harms, and considering other relevant evidence	p. 17-19
**Other information**			
Registration	23	Registration number and name of trial registry	p. 3
Protocol	24	Where the full trial protocol can be accessed, if available	https://pubmed.ncbi.nlm.nih.gov/30077178/
Funding	25	Sources of funding and other support (such as supply of drugs), role of funders	p. 22
